# The Organon, Section 153

**Published:** 1888-11

**Authors:** W. H. A. Fitz

**Affiliations:** Philadelphia


### THE ORGANON, SECTION 153.

"This search for a homœopathic, specific remedy, consists in the *comparison* of the totality of the symptoms of the natural disease with the lists of symptoms of our tested drugs, among which a morbific potency is to be found, corresponding in similitude with the disease to be cured. In making this comparison the more *prominent*, *uncommon*, and *peculiar* (characteristic) features of the case are especially, and almost exclusively considered and noted; *for these, in particular, should bear the closest similitude to the symptoms of the desired medicine,* if that is to accomplish the cure. The more general and indefinite symptoms, such as want of appetite, headache, weakness, restless sleep, distress, etc., unless more clearly defined, deserve but little notice on account of their vagueness, and also because generalities of this kind are common to every disease, and to almost every drug."

Mr. President and Gentlemen:—I have chosen the above section for discussion as its practical application in treating the sick determines the homœopathicity of the prescription, differentiates the homœopathic physician, and is the shortest cut to the cure of the disease.

'Tis easier to receive advice than to follow it, and I think this is excellently demonstrated by this section.

One would necessarily infer from this that all physicians would be in perfect accord as to the remedy when the teachings contained therein were properly applied to a given case. But is this true? Do physicians ever disagree as to what is the most similar remedy in this given case? In a large number of cases, *yes*. I propose to offer some points for discussion as the possible reason why there is such a confusion of opinion at the present time.

In a word the whole trouble is *Education.*

In following the advice of the section we must determine at the outset exactly what these *prominent, uncommon*, and *characteristic* features of the case are. What is the best means to attain this end, how shall we educate ourselves to make this practicable and of easy solution, when ignorance or doubt is all we possess?

If any one wish to study medicine, his first idea is to select a preceptor. This selection is almost as vital to his future success as the proper remedy is to the patient. Early education is generally a life-long accompaniment. If he wish to be a purist his paramount object should be to select one whose practice shows his principles. Having thus begun, he should read and study the *Organon.* After having understood its principles, his preceptor should review its study with him, emphasizing the points by practical suggestions, and the study of cases, old and new, to impress its philosophy. The student soon finds his knowledge is very limited with regard to the body whose ills he expects to know. He now begins his regular work in Anatomy, Physiology, Chemistry, Practice, etc., all with one end in view—how to detect and cure those ills. The College thus comes in to supplement the work already begun. Here is the next most important, as well as dangerous, step in his existence. What guarantee has he that his education will be in thorough harmony with the *Organon?* In the clinics the Professors examine the cases all through his course, thus depriving him of that most essential requirement in practice—the thorough examination of the patient. Instead of being required to suggest and analyze the remedies that have the most bearing upon the case, the symptoms and remedy are both given, and he is again deprived of the next most important point in practice—comparison of the symptoms of the *remedy* with those of the *case.*

When in doubt, after graduation, as to the remedy in a case from whose imperfect examination he had not been able to elicit the trite characteristics he has so often heard in college, he seeks the advice of a brother physician. Usually from every one to whom he applies he receives a different suggestion. What is he to do with the advice of "section 153" now? Can six or seven answers to the same question be true, when Hahnemann distinctly points out in section 118 that each remedy has an individuality which cannot be re-produced by another?

What is to be done? His patient needs attention, his living depends upon his clientage. He becomes a make-shift, adopting anything that offers the faintest ray of hope or success. This *habit* soon becomes *practice*, and from the promising student a full-fledged mongrel is matured. Is this an ideal picture? "He that runs may read." This is not all; the few who do bear the standard, are falsely adjudged from the practices of this pretender, and the school misrepresented.

It is much harder to-day to be a homœopath than formerly, for the reason that we have not kept system abreast of the means. The materia medica has increased, but where do we find the analytical repertory as the key to unlock its secrets?

There is a crying need for more *practical* training in our colleges. The last year should be especially devoted to applying clinically what has been learned theoretically. If three years be insufficient, four or five should be required. It is not numbers, but quality that is wanted in men who have human life as their stock in trade. Whoever heard of a boy listening to, or reading lectures for a period of years, and then setting himself up as a plumber, wheelwright, carpenter, or what-not, without ever having handled the very tools he must daily employ? Must medicine, the greatest of all professions, be an exception? A year well spent in examining and treating under a well-disciplined clinician, would sow seeds that would, in a few years, bear renown to the physician, his school and Alma Mater.

It is common for those students who can afford it, to go abroad for clinical instruction. This being *practical* work, it soon becomes the *habit* of the recipient, and in time he returns full of the latest crazes, and all the attendant paraphernalia to combat *germs;* with fine ability, probably, to diagnose the pathological lesion, but where are his methods of cure, his *diagnosis* of the *remedy*, his *Homœopathy?* This from want of impressive education and disuse, he has almost forgotten; he knows its theory and that is all.

He begins to practice, and what do we find—one who extols its methods? His practice gives the answer. Just here lies a wholesome lesson for the fraternity and all colleges.

Preceptors, select proper students. Colleges, extend your course, and above all incorporate clinical training in the curriculum under a supervising corps of teachers, and see to it that these men know, teach, and prescribe with the *Organon* as the expounder of their principles. Make better doctors, engender confidence by fidelity to principles, and reap the reward of your labors.

As the current practice of to-day is a so-called Homœopathy, not Hahnemannian, can we regain the lost ground and progress? We think so, but it behooves every one to face the responsibilities and work.

How shall we proceed?

1st. Each and every one *study* the *Organon*, to inculcate and perpetuate orthodoxy.

2d. Each one prove and study one or more drugs as Hahnemann directs in sections 105 to 145 inclusive, to educate our analysis, and create faith in the sick-making and curing power of drugs.

3d. Examine patients with the fidelity indicated in sections 85 to 105 inclusive, to show *why* the remedy was given, to discipline our observation and cull the chaff from the wheat for elimination from the materia medica.

4th. Join a society which makes materia medica and therapeutics its chief topic of discussion, to receive new ideas and assist our co-laborers with our experience.

5th. From study and experience suggest the characteristics of a remedy and prepare a repertory which gives its detailed analysis, with the concomitants of all these characteristics, to unify the means of finding *the* remedy.

By such concentration we shall be able to trace through the labyrinth of to-day a path which will lead to the goal of Truth so deeply hidden in "section 153."

W. H. A. Fitz, M. D.

Philadelphia.

